# The QP509L and Q706L superfamily II RNA helicases of African swine fever virus are required for viral replication, having non-redundant activities

**DOI:** 10.1080/22221751.2019.1578624

**Published:** 2019-02-19

**Authors:** Ferdinando B. Freitas, Gonçalo Frouco, Carlos Martins, Fernando Ferreira

**Affiliations:** CIISA – Centre for Interdisciplinary Research in Animal Health, Faculty of Veterinary Medicine, University of Lisbon, Lisbon, Portugal

**Keywords:** African swine fever virus, superfamily II RNA helicases, qRT-PCR, siRNA

## Abstract

African swine fever virus is complex DNA virus that infects pigs with mortality rates up to 100% leading to devastating socioeconomic effected in the affected countries. There is neither a vaccine nor a treatment to control ASF. African swine fever virus genome encodes two putative SF2 RNA helicases (QP509L and Q706L). In the present study, we found that these two RNA helicases do not share a common ancestral besides sharing a sequence overlap. Although, our phylogenetic studies revealed that they are conserved among virulent and non-virulent isolates, it was possible to observe a degree of variation between isolates corresponding to different genotypes occurring in distinct geographic regions. Further experiments showed that QP509L and Q706L are actively transcribed from 4 h post infection. The immunoblot analysis revealed that both protein co-localized in the viral factories at 12 h post infection, however, QP509L was also detected in the cell nucleus. Finally, siRNA assays uncover the relevant role of these proteins during viral cycle progression, in particular, for the late transcription, genome replication, and viral progeny (a reduction of infectious particles up to 99.4% when siRNA against QP509L was used and 98.4% for siRNA against Q706L). Thus, our results suggest that both helicases are essential during viral infection, highlighting the potential use of these enzymes as target for drug and vaccine development against African swine fever.

## Introduction

African swine fever (ASF) was first described in 1921 by Montgomery [1], being caused by African swine fever virus (ASFV) a large (≈200 nm), enveloped, icosahedral double-stranded DNA virus, which belongs to the NCLDV order and *Asfarviridae* family [2]. In domestic pigs, the ASFV replicates, preferentially, in cells of the monocyte lineage causing a broad range of symptoms and lesions, ranging from hyperacute to chronic forms of disease, with mortality rates up to 100%. Therefore, ASF leads to devastating effects on pig production and animal trade with high economic and social costs to affected areas [3,4].

Besides being endemic in most sub-Saharan countries and in Sardinia, ASF was introduced in Georgia (2007) spreading to neighbour countries including Armenia, Azerbaijan, Russia [5,6], and Ukraine and Belarus (from 2012 to 2013). In 2014, ASF was reported in Lithuania, making the first arrival of the disease in European Union in decades, before outbreaks in Poland, Estonia, and Latvia [7]. During 2016, ASF was declared in Moldova and last year in Czech Republic, Romania, Hungary, and Belgian (August 2018) putting the European Union on high alert. Also during this year, and for the first time, ASF was identified in several cities of China [8]. Since neither, a vaccine nor a treatment is available, the control of the disease relies on sanitary measures, including stamping out and trade bans of animals and pork products.

Under this scenario, further studies are needed towards the identification of ASFV genes that regulate viral replication and transcription, in order to develop an efficient vaccine and/or to use as targets for antiviral agents [9,10]. In other virus, RNA helicases have been described as essential for infection, modulating RNA–RNA and RNA–protein interactions, gene expression, viral egress, and host antiviral responses [11,12], being used for novel antiviral strategies [11,13,14]. Interestingly, ASFV encodes for five putative RNA helicases, including the DEAD-box ATP-dependent RNA helicases QP509L and Q706L [9–12]. Although *in silico* analysis revealed that QP509L is orthologous to the Vaccinia virus A18R helicase [15–17] and Q706L to the Vaccinia virus D6/D11 helicase [15,18], no additional information is available on these viral enzymes. Therefore, in this study, we investigated the monophyly of the five RNA helicases encoded by ASFV and explore the phylogenetic relationship of the QP509L and Q706L among different ASFV isolates and with DEAD-box ATP-dependent RNA helicases from other nucleocytoplasmic large DNA viruses (NCLDV) [19]. The dynamics of the transcription and expression patterns of ASFV-QP509L and ASFV-Q706L RNA helicases were evaluated during the infection, as well as their intracellular distribution. Finally, the involvement of each ASFV RNA helicases in viral transcription, genome replication, and progeny production was assessed by siRNA-mediated silencing.

## Results

### The ASFV DEAD-box RNA helicases QP509L and q706l are conserved among virulent and non-virulent isolates, uncovering genotype clustering and showing partial homology with RNA helicases of other NCLDV

**Figure 1.**
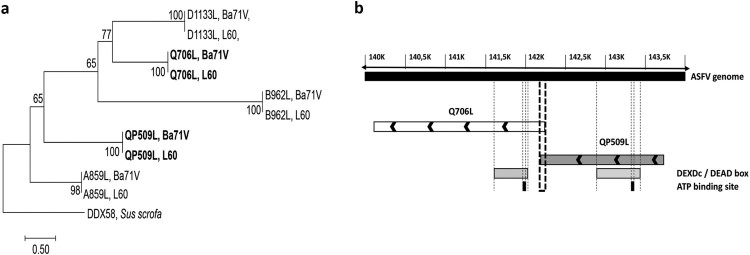
The ASFV-QP509L and Q706L RNA helicases are highly conserved among virulent and non-virulent isolates, sharing a distinct ancestor. (a) Phylogenetic analysis of ASFV RNA helicases. Maximum-likelihood phylogenetic tree constructed from a multiple nucleotide sequence alignment of ASFV-QP509L, Q706L, A859L, B962L, D1133L ORFs, using two different ASFV isolates (L60 – virulent and Ba71V – non-virulent). Sequence from a RNA helicase SF2 of *Sus scrofa* worked as outgroup. Bootstrap values are indicated. (b) Schematic representation of ASFV genome, including the localization of ASFV-QP509L and ASFV-Q706L. Relative positions of DEAD-box motifs, ATP-binding sites and sequence overlap (126 bp) are represented.

**Figure 2.**
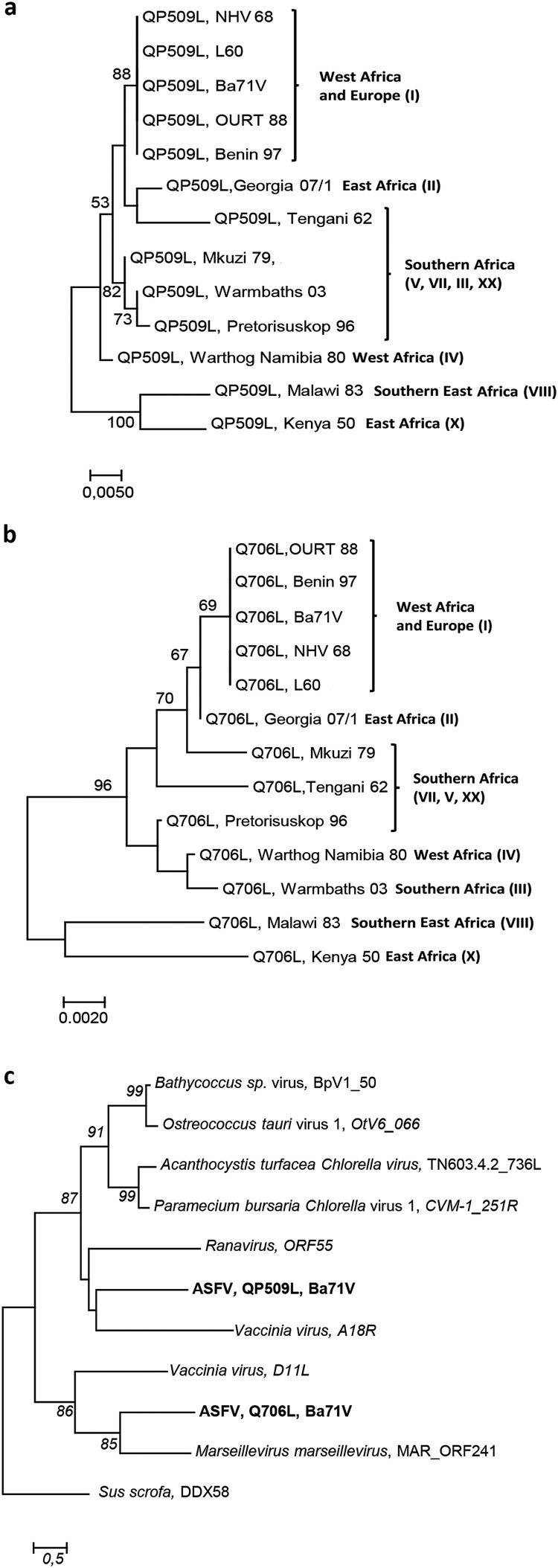
ASFV-QP509L and ASFV-Q706L RNA helicases show a similar genotype cluster segregation to ASFV-B646L, sharing the same monophyletic groups with other SF2 RNA helicases from NCLDV. (a) The maximum-likelihood phylogenetic tree was constructed from a multiple amino acid sequence alignment of ASFV-QP509L, using 13 different ASFV isolates. (b) Maximum-likelihood phylogenetic tree was constructed from a multiple amino acid sequence alignment of ASFV-Q706L, using 13 different ASFV isolates. Geographic distribution and genotype of ASFV isolates are also indicated. (c) Phylogenetic analysis of ASFV-QP509L, ASFV-Q706L and other viral Superfamily 2 (SF2) RNA helicases. The maximum-likelihood tree was generated from a multiple amino acid sequence alignment of ASFV-QP509L and ASFV-Q706L RNA helicases with other SF2 encoded by NCLDV members. The sequence of one SF2 RNA helicase of *S. scrofa* (DDX58) acted as outgroup. Bootstrap values are indicated.

The sequence homology analysis among the five ASFV RNA helicases revealed a high degree of similarity between virulent and non-virulent ASFV isolates (e.g. L60 and Ba71V, Figure 1(a)). Our analysis also showed that ASFV RNA helicases do not share a common ancestor, with the exception of ASFV-Q706L and ASFV-D1133L helicases that form a monophyletic group (Figure 1(a)). Surprisingly, no phylogenetic relation was found between ASFV-QP509L and ASFV-Q706L, although belonging to the Super family 2 and sharing a DEAD-box domain and a sequence overlap of 126 bp (between 3′ end of ASFV-QP509L and 5′ end of ASFV-Q706L, Figure 1(b)). Furthermore, the phylogenetic analysis of both SF2 RNA helicases among different ASFV isolates, showed a similar cluster distribution, with sequences from the West of Africa and Europe (e.g. Angola, L60, Ba71V) being separated from isolates of East Africa countries (e.g. Kenya 1950 and Malawi 1983) and also from South African isolates (e.g. Mkuzi 1979) (Figure 2(a,b)). In addition, the sequence of ASFV-QP509L RNA helicase from the European isolate Georgia 2007/1 clusters with the Tengani 1962 (Malawi) isolates (Figure 2(a)), whereas the ASFV-Q706L sequence from Georgia 2007/1 appears isolated (Figure 2(b)). Finally, the comparison of the amino acid sequences between the two SF2 RNA helicases encoded by ASFV and SF2 RNA helicases encoded by other NCLDV members revealed that ASFV-QP509L clusters with the A18 helicase from Vaccinia virus and with ORF55 Ranavirus helicase, as ASFV-Q706L clusters with the D11 helicase from Vaccinia virus and MAR_ORF241 helicase of Marseillevirus (Figure 2(c)). This analysis also showed that ASFV-QP509L sequence is more close to other viral SF2 RNA helicases encoded by other NCLDV than ASFV-Q706L (e.g. Bathycoccus sp. RCC1105 virus; Ostreococcus tauri; Chlorella virus 1) (Figure 2(c)).

### QP509L and Q706 ASFV genes are transcribed during infection, encoding for two intermediate-late proteins with distinct localization

**Figure 3.**
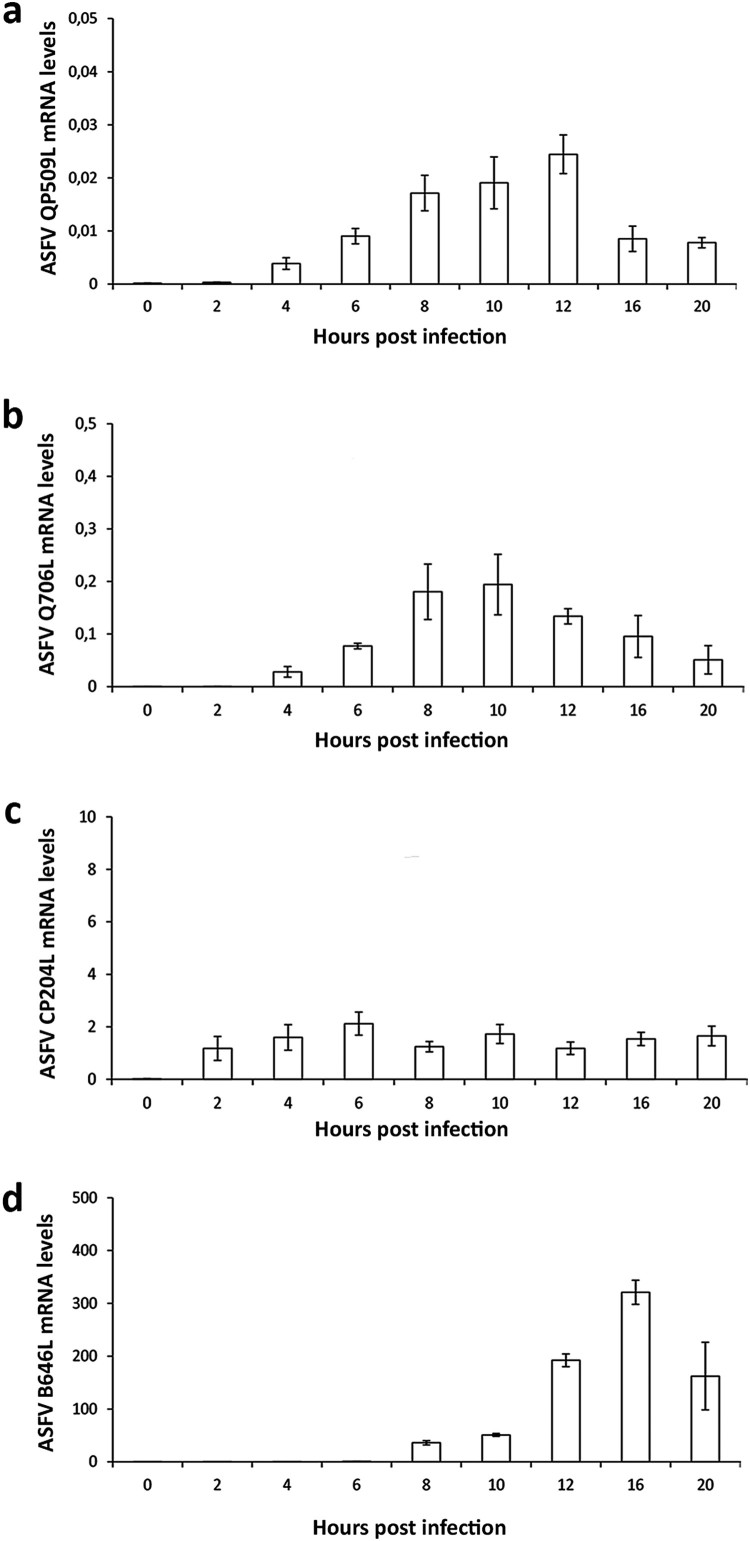
ASFV-QP509L and ASFV-Q706L are transcribed from early times of infection. (a) ASFV-QP509L transcripts were detected from 2 hpi, showing a maximum concentration peak at 12 hpi. (b) ASFV-Q706L transcripts were detected from early times of infection (2 hpi) reaching a maximum concentration at 10 hpi. (c, d) ASFV-CP204L and ASFV-B646L mRNA were used as controls. Results are shown as mean ± standard error of the number of transcripts of each viral gene normalized with Cyclophilin A mRNA levels (reference gene). Three independent experiments were performed in duplicate.

The detection and quantification of specific viral transcripts by qPCR revealed that QP509L gene is actively transcribed from 2 hpi onwards, reaching a maximum concentration peak at 12 hpi (Figure 3(a)). Similarly, the ASFV-Q706L transcripts were detected from 2 hpi, showing a peak concentration at 10 hpi (Figure 3(b)). As expected, the mRNA levels of both ASFV SF2 RNA helicases were found much lower than the transcripts of two viral genes (CP204L and B646L) which encode for two structural proteins (vp32 and vp72, Figure 3(c,d)), suggesting that expression of the two ASFV RNA helicases is highly regulated.

**Figure 4.**
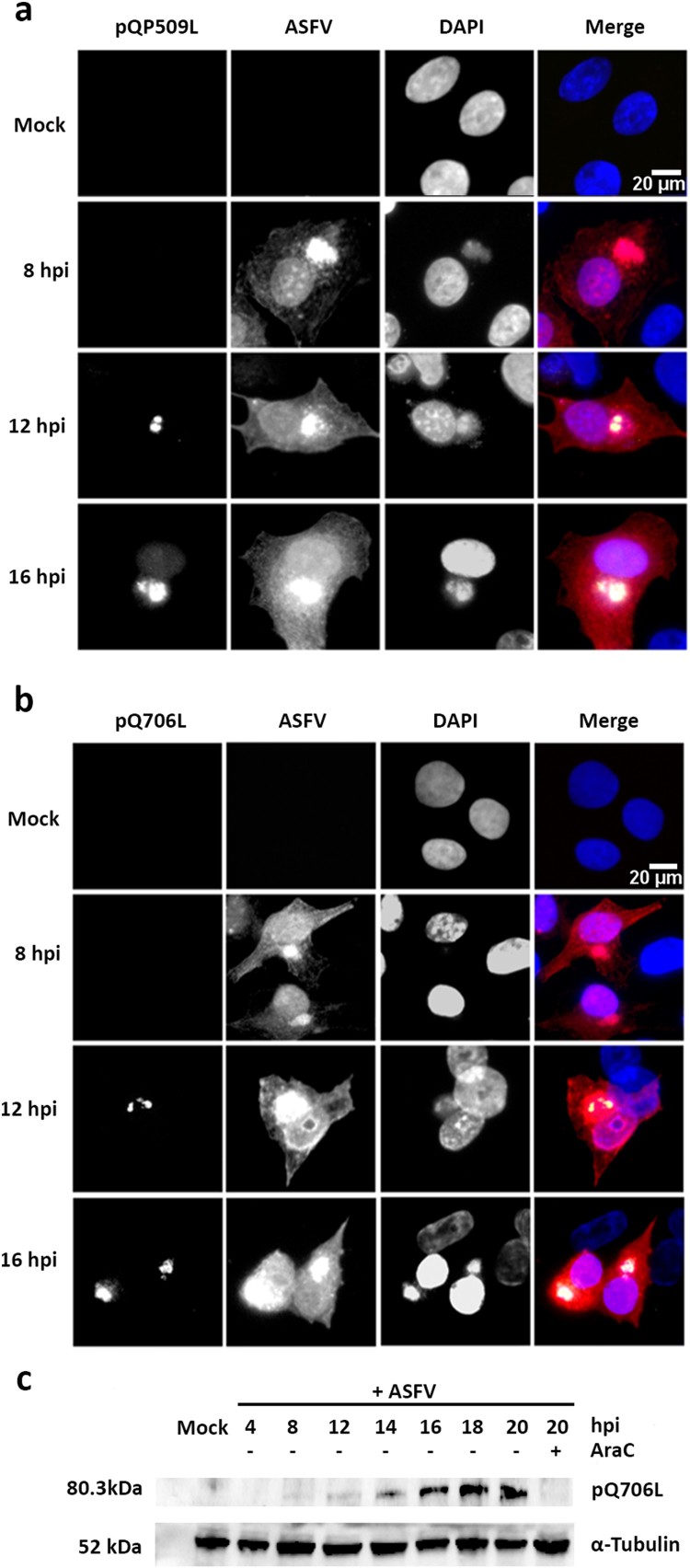
ASFV-pQP509L and ASFV-pQ706L are detected at late times of infection, showing distinct distribution patterns. (a) ASFV-pQP509L was detected at viral factories and host nucleus from 12 hpi onwards. (b) ASFV-pQ706L was identified only within viral factories and also after 12 hpi. Vero-infected cells (MOI = 2) were fixed (4, 8, 12, and 16 hpi), stained and analysed by fluorescence microscopy. In the merged images, ASFV-pQP509L and ASFV-pQ706L were labelled in green, infected cells in red and DNA in blue (DAPI staining). Representative images of at least three independent experiments are shown. (c) Immunoblot analysis revealed that ASFV-pQ706L is a late protein, being absence in the presence of AraC. Vero cells infected with ASFV/Ba71V isolate (MOI of 5) were harvested at the indicated time points. The cytosine arabinoside exposure (AraC, 50 µg/ml) was performed after an initial viral adsorption period (1 h) and cells were collected at 20 hpi.

In parallel, immunostaining studies showed that pQP509L accumulates in viral cytoplasmic factories from 12 hpi onwards, displaying a diffuse nuclear localization at later times of infection (Figure 4(a)). Regarding pQ706L, results disclosed that this viral protein is also detected from 12 hpi onwards, without any nuclear staining (Figure 4(b)). The immunoblot analysis unveiled that pQ706L is detectable from 12 hpi onwards, showing increased concentrations among infection course, corroborating the immunostaining results (Figure 4(c)). Finally, pQ706L expression was absent in ASFV-infected cells exposed to cytosine arabinoside (AraC), an inhibitor of ASFV DNA replication and late transcription (Figure 4(c)), suggesting that ASFV-Q706L RNA helicase is synthesized before viral DNA replication.

### QP509L and q706l ASFV RNA helicases are required for viral infection showing non-redundant functions

**Figure 5.**
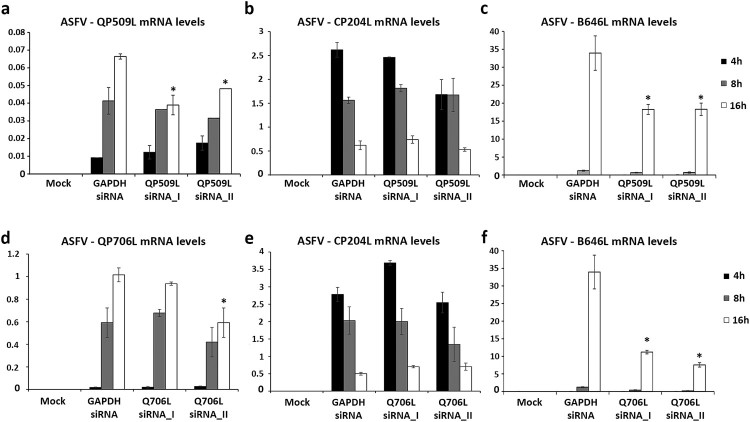
siRNAs targeting ASFV-QP509L and ASFV-Q706L transcripts disrupt late viral transcription. (a) siRNAs against ASFV-QP509L showed significant depletion efficacy at 16 hpi (*p* ≤ 0.05). (b) Unchanged ASFV-CP204L mRNA levels between QP509L-depleted cells and control group. (c) QP509L-depleted cells showed significant lower mRNA levels of late ASFV-B646L gene (*p* ≤ 0.05). (d) Q706L siRNA_II showed significant knockdown efficacy at 16 hpi (*p* ≤ 0.05). (e) Unchanged ASFV-CP204L mRNA levels between Q706L- knockdown cells and control group. (f) Q706L-depleted cells showed significantly lower mRNA levels of ASFV-B646L gene (*p* ≤ 0.05). Results are shown as average ± standard error (AVG ± S.E.), between the number of molecules of each viral transcript and the number of Cyclophilin A mRNA molecules (reference gene). Three independent experiments were performed in duplicate.

Taking in the consideration, the expression patterns of both viral SF2 RNA helicases, siRNAs experiments were performed to explore the downregulation effect of ASFV-pQP509L or ASFV-pQ706L in viral replication. To achieve this goal, the efficacy of two siRNAs targeting each ASFV transcript was separately assed. A significant knockdown efficiency was found for both siRNAs targeting ASFV-QP509L transcripts (up to −41.2%, *p* ≤ 0.05, Figure 5(a)) and for a siRNA duplex against ASFV-Q706L transcripts (up to −41.7%, *p* ≤ 0.05, Figure 5(d)). Additional qPCR analysis revealed lower transcription levels of ASFV B646L late gene in ASFV-QP509L depleted cells (up to −46.2%, *p* ≤ 0.05, Figure 5(c)) and in ASFV-Q706L-knockdown cells (up to −77.7%, *p* ≤ 0.05, Figure 5(f)) in comparison to control infected cells (transfected with siRNA targeting GAPDH transcripts). However, no significant reduction in the transcriptional activity of ASFV CP204L early gene was found (Figure 5(b,e)).

**Figure 6.**
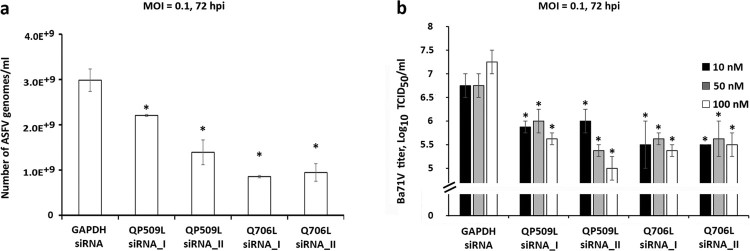
ASFV-QP509L and ASFV-Q706L downregulation disrupts ASFV DNA replication and progeny production. (a) QP509L and Q706L-depleted cells showed a decreased number of ASFV genomes [2.21 × 10^9^ genomes/ml using QP509L siRNA_I (−26%), 1.39 × 10^9^ genomes/ml for QP509L siRNA_II (−53%), 8.53 × 10^8^ genomes/ml for Q706L siRNA_I (−71%) and 9.45 × 10^8^ genomes/ml using Q706L siRNA_II (−68.32%)] when compared to the control group (2.98 × 10^9^ genomes/ml, *p* ≤ 0.05). Results represent the mean of three independent experiments. (b) A statistically significant reduction in viral yields was observed between ASFV-infected Vero cells (MOI of 0.1) transfected with siRNAs against ASFV-QP509L and ASFV-Q706L (−82.2% and −99.4% for QP509L siRNA I and II, respectively, and −92.5% and 98.6% for Q706L siRNA I and II), in comparison with the GAPDH siRNA-transfected infected cells (*p* ≤ 0.05). Results were obtained from three independent experiments. Error bars represent standard error (SE) of the mean values.

Additionally, a significant decreased number of viral genomes was measured in depleted cells, ranging between −26.1% and −53.4% in ASFV-QP509L-knockdown cells (*p* ≤ 0.05; Figure 6(a)) and between −68.3% and −71.4% in ASFV-Q706L-knockdown cells (*p* ≤ 0.05; Figure 6(a)). Finally, a significant reduction of viral progeny was found in ASFV-QP509L depleted cells (between −82.2% to −99.4%; *p* ≤ 0.05) and in ASFV-Q706L depleted cells (−92.5% to −98.6%) in comparison to controls (*p* ≤ 0.05, Figure 6(b)).

## Discussion

RNA helicases are found in all kingdoms of life, participating in several aspects of RNA metabolism and in different events of DNA replication [20,21]. Although, many viruses hijack cellular RNA helicases[20], members of some viral families encode their own, as *Herpesviridae* [22], *Poxviridae* [23], *Parvoviridae* [24], *Flaviviridae* and *Asfaviridae* [25]. Notably, ASFV encodes five putative RNA helicases, including the two pQP509L and pQ706L SF2 DEAD-box RNA helicases [26,27]. In eukaryotes, these enzymes are known to unwind duplexes formed during RNA transcription, in an ATP-dependent fashion [28–30], with viral counterparts being involved in DNA–RNA and RNA–protein interactions that occur from the beginning of viral gene expression and culminate with the release of infectious particles [11,12]. Thus, these proteins are being explored as antiviral drug targets [14]. In ASFV, and besides the initial sequence data assembly and annotation, scarce information is available about the role of viral RNA helicases in infection. In this study, we showed that ASFV RNA helicases are highly conserved among virulent and non-virulent isolates, with ASFV-QP509L and ASFV-Q706L helicases belonging to distinct monophyletic lines, despite their partial sequence overlap and common superfamily 2 motifs. An identical geographic/genotype cluster segregation was found for both viral RNA helicases, similar to the one reported for ASFV-B646L [31–33] and other viral genes [34]. However, probably due to the recent recombination events reported in Georgia 2007/1 isolate [35], the QP509L sequence of this genotype II clusters with an ASFV isolate belonging to genotype V (Tengani 62), reinforcing the idea that viral phylogenetic studies should include more than one ORF [36]. In addition, the comparison between the two ASFV SF2 RNA helicases and SF2 RNA helicases encoded by other NCLDV members revealed that ASFV-QP509L shares the same monophyletic group with *Vaccinia* A18R and *Ranavirus* ORF55 RNA helicases, whereas ASFV-Q706L clusters with *Vaccinia virus* D6/D11L and *Marseillevirus marseillevirus* MAR_ORF241 RNA helicases, corroborating previous studies [15–18,37]. Although these results are somehow expected, since NCLDV members share a common ancestor [19], the two genes of SF2 RNA helicases in Vaccinia virus are separated approximately 20000 bp, suggesting a different evolutionary route for ASFV. Indeed, recent studies reported that ASFV presents a higher evolutionary rate than other DNA virus [37–39], probably due to its complex inter-species transmission (wild boars, ticks, and domestic pigs), showing a diversity peak over the last 200 years [39].

**Figure 7.**
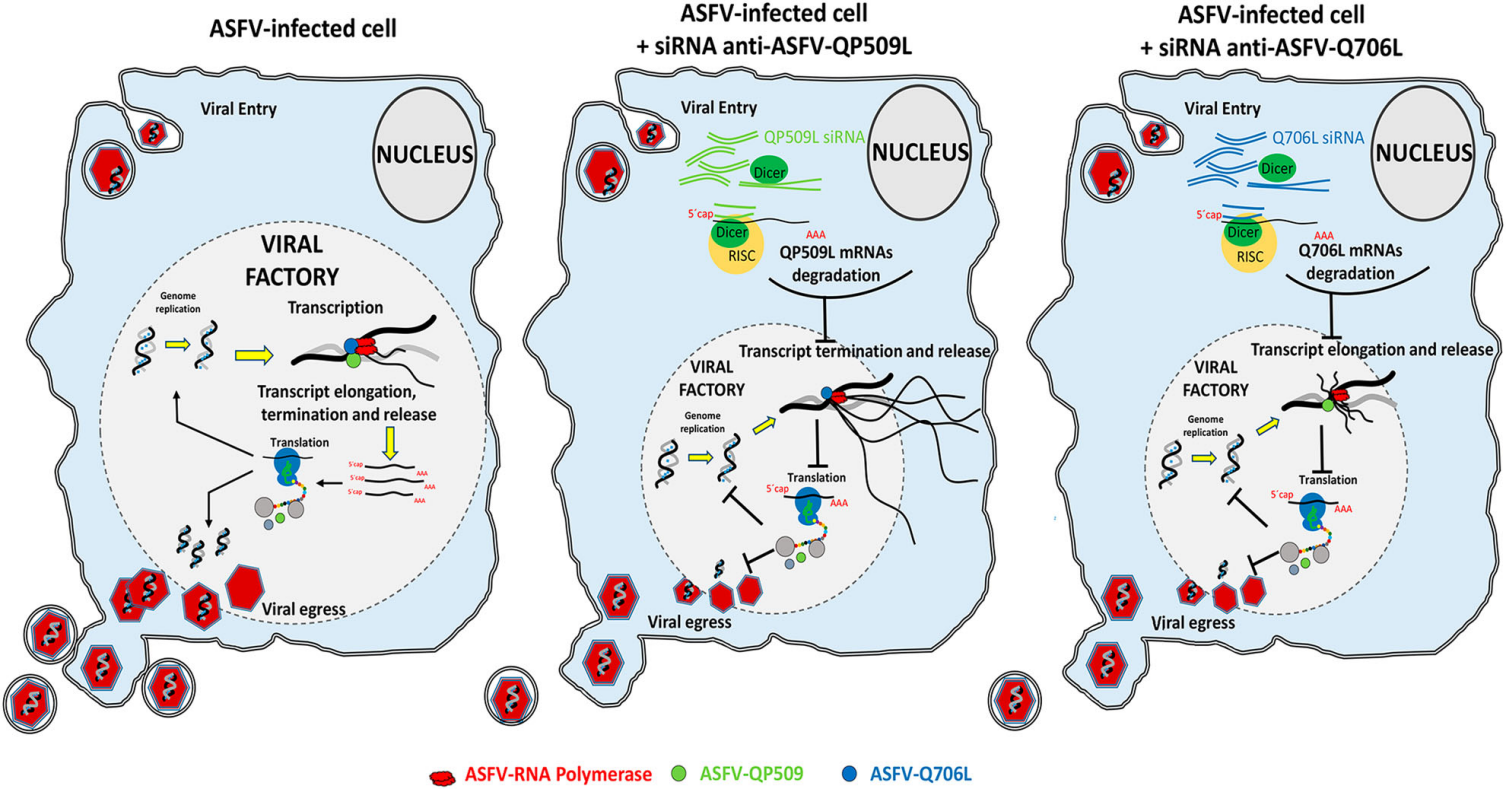
Proposed working model for ASFV-QP509L and ASFV-Q706L RNA helicases. Considering the experimental data available for ASFV-QP509L and ASFV-Q706L orthologous (A18R and D6/D11 SF2 RNA helicases of Vaccinia virus, respectively), our model hypothesized that absence of ASFV-QP509L on the transcription complex will lead to continuous reading of neighbouring viral ORFs and transcriptionally silenced regions, giving rise to an accumulation of long RNAs, with its release being also affected. In parallel, our working model postulates that downregulation of ASFV-Q706L will disrupt transcription elongation and termination with serious implications in viral progeny production.

Regarding the expression patterns of two ASFV SF2 RNA helicases, maximum mRNA levels were detected between 8 and 12 hpi, suggesting that both enzymes are mainly required during the intermediate and late stages of the infection cycle, when the viral DNA replication and transcription are more active. In fact, pQP509L was detected from 12 hpi within viral factories and host nucleus, whereas, pQ706L was detected only at viral factories from 12 hpi onwards, indicating that both ASFV RNA helicases have different roles during replication cycle. Despite an early intranuclear phase has been proposed for ASFV [40,41], the presence of pQP509L in this cellular compartment, at later times of infection, can be related to other viral events than transcription and/or DNA replication as, for example, modulation of antiviral responses. This plethora of activities is described for other viral RNA helicase as, for example, in NS3 RNA helicase of Hepatitis C virus (HCV) which is involved in unwinding of the double-stranded RNA intermediates [42] and viral assembly [43], and in D6/D11 RNA helicase of Vaccinia virus that unwinds RNA–RNA, RNA–DNA, and RNA–protein intermediates [23]. Thus, these viral SF2 DEAD-box RNA helicases were described as essential for infection by HCV [44,45], Vaccinia virus [13,46], and Plum pox virus [47], with cellular RNA helicases being unable to rescue the activity of viral counterparts. In a very similar way, our results from siRNA experiments disclosed that QP509L- and Q706L-knockdown cells show lower levels of late viral transcripts (ASFV-B646L), although the expression of an early viral gene (ASFV-CP204L) is not affected. Depleted cells also exhibited a reduced number of viral genomes coupled with a decreased viral yield, indicating that both ASFV RNA helicases have relevant and non-redundant functions, not rescued by cellular RNA helicases. Considering our above results and the data reported on Vaccinia virus counterparts, a working model for both ASFV SF2 RNA helicases is depicted in Figure 7. Briefly, ASFV-QP509L and ASFV-Q706L RNA helicases are mainly involved in viral transcription events, with ASFV-QP509L assisting termination and release of late viral transcripts (as reported for Vaccinia virus A18R helicase orthologous), whereas ASFV-Q706L regulates elongation and release of late viral transcripts (as Vaccinia virus D6/D11 helicase).

Finally, taking into consideration that there is neither a vaccine nor a treatment available against ASFV and the important roles of both ASFV SF2 RNA helicases, we hypothesized that a mutant on ASFV-QP509L or ASFV-Q706L gene can be a good candidate to generate a live attenuated vaccine. These ASFV mutants will not produce progeny, allowing the immediate-early and early viral gene expression and providing antigens that can induce a protective immune response.

## Material and methods

### Phylogenetic analysis

Amino acid sequences of the five ATP-dependent RNA helicases of ASFV (QP509L, Q706L, A859L, B962L and D1133L) were obtained by *in silico* translation using genomic sequences of different ASFV isolates available in GenBank (ASFV/Ba71 V, NC_001659.1; ASFV/Benin 97/1, AM712239.1; ASFV/L60, KM262844.1; ASFV/OURT 88/3, AM712240.1; ASFV/Mkuzi 1979, AY261362.1; ASFV/Georgia 2007/1, FR682468.1; ASFV/Malawi Lil/20/1, AY261361.1; ASFV/Kenya 1950, AY261360.1; ASFV/NHV 1968, KM262845.1; ASFV/Tengani 62, AY261364.1; ASFV/Warmbaths 2003, AY261365.1; ASFV/Pretorisuskop/96/4, AY261363.1; ASFV/Warthog 2004, AY261366.1). Similarly, the amino acid sequences of other superfamily 2 (SF2) RNA helicases encoded by different NCLDV (*Ranavirus,* ORF55, ASQ42908.1; *Bathycoccus sp*. RCC1105 virus BpV1, BpV1_050, NC_014765.1; *Acanthocystis turfacea Chlorella* virus, TN603.4.2_736L, JX997186.1; *Paramecium bursaria Chlorella* virus CVM-1, CVM-1_251R, JX997163.1; *Vaccinia* virus, A18R, NC_006998.1; *Vaccinia* virus, D11, NC_006998.1; *Marseillevirus marseillevirus*, MAR_ORF241,NC_013756.1; *Ostreococcus tauri* virus, OtV6_066, JN225873.1) and by pig (*Sus scrofa,* DDX58, AAG09428.1) were also retrieved from GeneBank.

MAFFT software (version 7, https://mafft.cbrc.jp/alignment/server/) was used to perform sequence alignments (employing default parameters) [48,49] and MEGA 7 *software* was used to select the best model (ML) for phylogenetic tree construction based on amino acid alignments [50,51]. Maximum-likelihood trees were constructed by adopting the Le_Gascuel_2008 model with 1000 bootstrap replicates, using MEGA 7 [51,52].

### Cells and viruses

Vero E6 cells obtained from the European Authenticated Cell Cultures Collection (ECACC, Salisbury, UK) were maintained in DMEM (Dulbecco Modified Eagle’s minimal essential medium) supplemented with L-GlutaMax, 10% heat-inactivated foetal calf serum, 1x non-essential amino acids and penicillin/streptomycin at 100 units/ml (all from Gibco, Life Technology, Karlsruhe, Germany). All experiments were conducted on actively replicating sub confluent cells, grown at 37°C, under a 5% CO_2_ humidified atmosphere (≥95% air). The Vero-adapted ASFV strain (Badajoz 1971, Ba71 V) was used in all infections and propagated as previously described [53]. For viral titration, culture supernatants harvested at 72 h post infection (hpi) were used to infect new cell monolayers, in 10-fold serial dilutions, during 5 days (Spearman–Kärber endpoint method) using 96-well plates. Cytopathic effect was evaluated and the results were expressed as TCID50/ml.

### RNA extraction and cDNA synthesis

For qPCR analysis, total RNA was extracted from ASFV-infected Vero cells (MOI = 1.5) at different time points of infection, using the RNeasy Mini Kit (Qiagen, Courtaboeuf, France) and with possible DNA contaminants degraded by a treatment in a column with DNAse I (Qiagen). RNA concentrations and purity were measured using a spectrophotometer (NanoDrop 2000c, Thermo Fisher Scientific, Waltham, USA) and only RNA samples showing high purity (A260/A280 ratio between 1.8 and 2.0) were used. 200 ng of each total RNA sample was reverse transcribed (Transcriptor First Strand cDNA Synthesis Kit, Roche, Basel, Switzerland), according to the manufacturer’s instructions. The obtained cDNA was diluted (1/20) in ultra-pure water and stored at −20°C until further use.

### Recombinant plasmids

The amplified fragments (ASFV-QP509L, ASFV-Q706L, ASFV-CP204L, ASFV-B646L, and Cyclophilin A) were cloned into a plasmid vector (pGEM-Teasy Vector System II, Promega, Madison, USA), and used to transform DH5α competent cells. Then, plasmids were isolated from bacteria using the Roche High Pure Plasmid Isolation Kit (Roche Applied Science, Germany), according to the manufacturer’s manual. To determine whether the cloned DNA fragments were incorporated into vectors, the inserts were amplified by PCR and their sequences were confirmed. Following this step, the concentration of each plasmid preparation was determined by spectrophotometric absorbance (NanoDrop 2000c). Their corresponding copy number was calculated using the equation: pmol (dsDNA) = μg (dsDNA) × 1515 / DNA length in pb (pmol = picomoles, dsDNA = double-strand DNA, DNA length in pb = number of base pairs from the amplified fragment; 1 mol = 6.022 × 10^23^ molecules).

### Standard curves optimization

Ten-fold serial dilutions of each plasmid (ASFV-QP509L, ASFV-Q706L, ASFV-CP204L, ASFV-B646L, and Cyclophilin A), ranging from 1 × 10^−1^ to 1 × 10^−9^ were initially used in duplicate to generate the standard curves in two different days. Threshold cycle (Ct) values obtained from each dilution were plotted against the logarithm of their initial template copy numbers and corresponding standard curves were generated by linear regression of the plotted points. From the slope of each standard curve, PCR amplification efficiency (E) was calculated according to the equation: E (%) = (10^−1^/slope^−1^) × 100% [54].

### Quantitative PCR

Quantification of ASFV-QP509L, ASFV-Q706L, ASFV-CP204L, and ASFV-B646L transcripts were performed by qPCR using the Maxima SYBR Green PCR Master Mix (Thermo Fisher), according to the manufacturer’s instructions [12.5 µl of master mix, 2.5 µl of forward and reverse primers (50 nM each), 5 µl of Milli-Q water and 2.5 µl of cDNA)]. All qPCR reactions were performed in Applied Biosystems 7300 Real Time PCR system (Thermo Fisher), using the following thermal profile: 10 min at 95°C for initial denaturation; 40 cycles of 15 s at 95°C and 60 s at 60°C, followed by a final denaturation step of 5 s at 65°C with a 20°C/s ramp rate and subsequent heating of the samples to 95°C with a ramp rate of 0.1°C/s. Quantification of ASFV-QP509L, ASFV-Q706L, ASFV-CP204L, ASFV-B646L, and Cyclophilin A mRNA levels were determined by the intersection between the fluorescence amplification curve and the threshold line. The crossing point values of each plasmid obtained from different known concentrations were plotted in a standard curve used to determine the copy number of each transcript. The values were determined using the comparative threshold cycle method, which compares the expression of a target gene normalized to the reference gene (Cyclophilin A). The validation of the reference gene was confirmed using the ANOVA test (*p* < 0.05) and the specificity of the qPCR assays was confirmed by melting curve analyses. Sequences of the primers used in this study are shown in Table 1. To characterize the transcription pattern of both ASFV RNA helicases, Vero cells grown onto 30 mm dishes were infected (MOI = 1.5) and collected at indicated time points (0, 2, 4, 6, 8, 10, 12, 16, and 20 hpi), for total RNA extraction. Results represent the mean value of two independent experiments performed in different days.

**Table 1.** Primers used in the present study.

Target	Primer designation	Sequence (5′–3′)	Target coordinates^a^	Orientation
**ASFV-QP509L**	509FwE	GTGCCTGAGAAAGAGCGGTA	142562–142581	Forward
**ASFV-QP509L**	509FwI	GTCCCACCACAACCTTTTCC	142927–142908	Forward
**ASFV-QP509L**	509RvI	AATACACACAGGGCTAACGAAGT	142870–142848	Reverse
**ASFV-Q706L**	706FwE	TCCCCGTCCAAATAGAAGCA	142211–142192	Forward
**ASFV-Q706L**	706FwI	CAGGGGGAAAACACACGGG	142050–142032	Forward
**ASFV-Q706L**	706RvI	AAGTGAGATGGCAAGCGACA	154439–154420	Reverse
**Cyclophilin A**	CycloFw1	AGACAAGGTTCCAAAGACAGCAG	–	Forward
**Cyclophilin A**	CycloRev	AGACTGAGTGGTTGGATGGCA	–	Reverse
**Cyclophilin A**	CycloFw2	TGCCATCCAACCACTCAGTCT	–	Forward
**ASFV-B646L**	VP72Fw	ACGGCGCCCTCTAAAGGT	88273–88290	Forward
**ASFV-B646L**	VP72Rev	CATGGTCAGCTTCAAACGTTTC	88322–88343	Reverse
**ASFV-CP204L**	VP32Rev	TCTTTTGTGCAAGCATATACAGCTT	108162–108186	Forward
**ASFV-CP204L**	VP32Fw	TGCACATCCTCCTTTGAAACAT	108228–108249	Reverse
**ASFV-QP509L**	509FwSacI1	GAGCTCATGGCTTACAATAATGCAGCGTG	143213–143194	Forward
**ASFV-QP509L**	509RvXhoI1	CTCGAGAGGGCTAACGAAGTCAGGA	142875–142857	Reverse
**ASFV-QP509L**	509FwSacI2	GAGCTCATGTACGGGCGTAGAGGCA	142529–142514	Forward
**ASFV-QP509L**	509RvXhoI2	CTCGAGTTTGGACGGGGAAGGA	142215–142200	Reverse
**ASFV-Q706L**	706FwNdeI1	CATATGATGTATGAAAGATTCTACACCGCTTATG	141540–141516	Forward
**ASFV-Q706L**	706RvXhoI1	CTCGAGTTTTAGCATGCGCACTATTTT	141108–141088	Reverse
**ASFV-Q706L**	706FwNdeI2	CATATGATGTCTAAAACGGGAGCTGAGG	140742–140724	Forward
**ASFV-Q706L**	706RvXhoI2	CTCGAGTTCGTAAAAGGTATAGCCTAATCCTAC	140241–140215	Reverse

^a^Primer coordinates are relative to Ba71 V sequence used has template for primer design.

### Cloning, expressing and purifying recombinant fragments of ASFV-QP509L and ASFV-Q706L

In order to produce antibodies against ASFV-QP509 and ASFV-Q706L, the hydrophobicity profile of both viral proteins was analysed to select two distinct hydrophilic regions in each ORF. Taking into account this information, specific primers were designed to include in the 5′and 3′, a restriction enzyme site to facilitate vector insertion [ASFV-Q706L, clone using NdeI (5′) and XhoI (3′); ASFV-QP509L, clone using SacI (5′) and XhoI (3′)] and the correspondent DNA fragments were amplified by PCR. The PCR reactions were performed as follows: 1x 98°C for 2′, 30x 98°C for 30″, 72°C for 1′10″ plus one extension step of 72°C for 10′. After size confirmation in agarose gel (1%), the fragments were purified and DNA concentration was quantified (NanoDrop 2000c). The DNA fragments were inserted in the pET24a expression vector (Novagen) in order to add a C-terminal 6xHis tag to allow purification. Two clones per ORF were sequenced to confirm eventual mutations and plasmids transformed into the *E.coli* strain BL21(DE3)-pLysS (Novagen) and grown in LB medium (10 g tryptone, 5 g yeast extract, 5 g NaCl, pH 7.2), supplemented with kanamycin (30 μg/ml) plus chloramphenicol (34 μg/ml), at 37°C, with shaking at 200 rpm, until the OD_600_ reached 0.1–0.2. Protein expression was induced by adding isopropyl-β-D-1-thiogalactopyranoside (IPTG, 1 mM, 5 h). After this step, bacterial cells were harvested by centrifugation (10,000*g* for 10 min, 4°C), and washed with sterile water. The pellet was resuspended in binding buffer (20 mM sodium phosphate, 500 mM NaCl, 20 mM imidazole, pH 7.4) and cells were lysed with a lysis solution (0.2 mg/ml lysozyme, 20 µg/ml DNAse and 1 mM PMSF) and sonicated for 5 × 5 min on ice (5 cycles, 70% amplitude). Lysates were then centrifuged at 3000*g* for 15 min and pellets were discarded. The extracts were thereafter filtered (0.45 µm syringe filter Rotilabo^®^, CarlRoth) and incubated with Ni Sepharose 6 Fast Flow slurry (GE Healthcare) for 1 h. The mixture was loaded onto a PD-10 column (GE Healthcare), washed with binding buffer solution (20 mM sodium phosphate, 500 mM NaCl, pH 7.4) containing increasing concentrations of imidazole (40, 60, and 80 mM), and the recombinant fragments of pQP509L and pQ706L were eluted with an elution buffer (20 mM sodium phosphate, 500 mM NaCl, 500 mM imidazole, pH 7.4). Finally, fractions were collected in low-binding tubes (Maxymum Recovery^®^ TM tubes, Axygen, Corning Life Sciences, Amsterdam, The Netherlands), analysed by SDS-PAGE and the recombinant proteins, purified under native conditions and stored at −80°C until further use.

### Antibody production

Briefly, young female mice (BALB/c, 4–6-week-old) were injected subcutaneously with 100 μg of each purified fragment of ASFV-pQP509L and ASFV-pQ706L, in a mixture with Freund’s complete adjuvant. Following the primary injection, two booster injections were administered at 2-week intervals. After 10 days from the date of the second booster injection, the total blood was collected and sera were aliquoted and stored at −20°C until further use. The specificity of the polyclonal antiserum was tested against purified recombinant ASFV-pQP509L and ASFV-pQ706L and whole infected-cells extracts.

### Immunofluorescence and microscopy analysis

Vero cells seeded on glass coverslips (1 × 10^5^/cm^2^) were mock-infected or infected with the ASFV Ba71V isolate (MOI of 1). At 8, 12, and 16 hpi, cells were fixed in 3.7% paraformaldehyde and HPEM buffer [25 mM HEPES (4-(2-hydroxyethyl)-1-piperazineethanesulfonic acid), 60 mM PIPES (piperazine-N,N′-bis 2-ethanesulfonic acid), 10 mM EGTA (ethylene glycol tetraacetic acid), and 1 mM MgCl_2_] for 15 min, at room temperature, and permeabilized with PBS/Tx-100 (0.5%, v/v) during 5 min. Following this step, cells were washed in PBS, blocked with PBST/BSA (3%, w/v) for 30 min and incubated with the appropriate primary and secondary antibodies. The immunostaining of ASFV-pQP509L and ASFV-pQ706L and ASFV-infected cells was achieved by incubation with two in-house primary antibodies: mouse anti-ASFV-pQP509L and anti-ASFV-pQ706L (1:10 in PBST, 0.01%, overnight, 4°C) and swine anti-ASFV polyclonal antibody (1:100, 1 h, RT). Two secondary fluorescent-conjugated antibodies were used as follows: anti-mouse FITC (1:300, sc-2099, Santa Cruz Biotechnology) and anti-swine Texas Red (1:500, ab6775, Abcam). Between each antibody incubation, cells were washed twice with PBS (5 min) and once with PBST (0.1% v/v, 5 min). All incubations were performed in a dark humidified chamber to prevent fluorochrome fading and a mounting medium with DAPI (4′,6-diamidino-2-phenylindole) was used to detect the cell nucleus and viral factories (Vectashield, Vector Laboratories, Peterborough, UK).

Fluorescence images were acquired using an epifluorescence microscope equipped with a 40× objective (Leica DMR HC model, Wetzlar, Germany) and data sets were acquired with the Adobe Photoshop CS5 software (Adobe Systems, Inc., San Jose, USA). Images were subsequently processed using the ImageJ open source software (version IJ 1.48 g, National Institutes of Health, Bethesda, MD, USA).

### Immunoblot analysis

Vero cells grown in 30 mm dishes were infected with the ASFV-Ba71 V isolate (MOI of 5) and when indicated, exposed to cytosine arabinoside (50 µg/ml, AraC; Sigma-Aldrich), after the adsorption period (1 h). Following this step and before protein extraction, mock-infected, infected, and AraC-treated infected-cells were washed twice with PBS and then lysed in ice-cold modified RIPA buffer [25 mM Tris, 150 mM NaCl, 0.5% (v/v) NP40, 0.5% (w/v) sodium deoxycolate, 0.1% (w/v) SDS, pH 8.2] supplemented with a protease-inhibitor cocktail (cOmplete, Mini, EDTA-free, Roche) and a phosphatase-inhibitor cocktail (PhosStop, Roche). Clarified whole-cell lysates harvested at 4, 8, 12, 14, 16, 18, and 20 hpi, were subjected to SDS-PAGE gel electrophoresis using 8–16% (w/v) polyacrylamide separating gels (Bio-Rad), and transferred to a 0.2 μm pore diameter nitrocellulose membrane (Whatman Schleicher & Schuell) by electroblotting. Blot membranes were then blocked with phosphate-buffered saline plus 0.05% (v/v) Tween-20 (PBST), containing 5% (w/v) of BSA (Sigma-Aldrich), during 1 h at RT, and thereafter incubated with specific primary antibodies (RT,1 h), followed by a wash step with PBST (3 × 10 min). The membranes were then incubated with appropriate secondary antibodies conjugated with HRP, for 1 h, at RT. Finally, a wash step in PBST (3 × 10 min) was performed before protein detection with a chemiluminescence detection kit (Pierce^®^ ECL Western Blotting Substrate, Thermo Scientific), on Amersham Hyperfilm ECL (GE Healthcare). α-Tubulin was used as a loading control. For the blot analysis, three primary antibodies (anti- ASFV-pQP509L and ASFV-pQ706L, 1:100; anti-α-tubulin, 1:1250, #2125, Cell Signalling Technology) and two HRP-conjugated secondary antibodies were used (anti-rabbit IgG, 1:10000, 4010–05; anti-mouse IgG, 1:30000, 1010–05; both from SouthernBiotech). All antibody dilutions were performed in blocking solution and incubated according to the manufacturers’ recommendations.

### siRNA assays

Four double-stranded siRNAs (ON-TARGETplus, Thermo Fisher Scientific) targeting different sequence regions of the ASFV-QP509L and ASFV-Q706L transcripts were designed (siDESIGN Center, Thermo Fisher Scientific), based on the full genome sequence of ASFV Ba71 V isolate (GenBank/EMBL, accession number: ASU18466). One siRNA against the GAPDH gene (siRNA-GAPDH; Silencer™ GAPDH siRNA human control number 4605; Ambion/Thermo Fisher Scientific) was used as a control in all siRNA assays. The siRNA sequences used in the study are shown in Table 2. All siRNAs duplexes were diluted at different final concentrations (10, 50, and 100 nM) in serum-free Opti-MEM (Gibco) and using 8 μl HiPerfect Transfection reagent (Qiagen). Mixtures were incubated at room temperature for 20 min to allow the formation of transfection complexes. Thereafter, 100 μl of the transfection solution was incubated with 2 × 10^4^ Vero cells cultured in 500 μl of DMEM supplement with 10% FBS in a 24-well plate, during 8 h. One hour before infection, the culture medium was removed and fresh medium was added to allow recovery of the cells. Next, cells were infected with ASFV Ba71V (MOI = 0.1) during one hour and harvested at 37°C for 16 hpi for quantification of the viral transcripts or 72 h for quantification of genome copy number and viral progeny titration. Due to economic and practical reasons, only the two siRNAs duplexes that showed higher inhibitory results (CPE reduction) were used for these assays. To ensure high RNA concentrations, the siRNA assays were performed in quadruplicated and the qPCR assay was performed in duplicate to improve the biological relevance of the results. The ASFV-genome copy number was estimated by measuring B646L gene using qPCR and TaqMan probes [55].

**Table 2.** siRNA sequences to knockdown ASFV-QP509L and ASFV-Q706L transcripts.

Target	siRNA designation	Sequence (5′–3′)	Target coordinates^a^	Orientation
ASFV-QP509L	QP509_IF	GCAAGAAGCCUGAGCAGUUUUUU	596–614	Sense
ASFV-QP509L	QP509_IR	AAAACUGCUCAGGCUUCUUGCUU	596–614	Anti-sense
ASFV-QP509L	QP509_IIF	AGCAAGAAAUGGUCGAUAAUUUU	302–320	Sense
ASFV-QP509L	QP509_IIR	AAUUAUCGACCAUUUCUUGCUUU	302–320	Anti-sense
ASFV-Q706L	Q706_IF	GGAUAAGGCCCGAGAGGAUUUUU	1566–1584	Sense
ASFV- Q706L	Q706_IR	AAAUCCUCUCGGGCCUUAUCCUU	1566–1584	Anti-sense
ASFV-Q706L	Q706_IIF	CCGAAAUGCUAACAGUAAAUUUU	1043–1061	Sense
ASFV- Q706L	Q706_IIR	AAUUUACUGUUAGCAUUUCGGUU	1043–1061	Anti-sense

^a^siRNA coordinates according to the relative position in gene nucleotide sequence (start at position 1, ATG).

### Statistical analysis

The GraphPad Prism software (version 7.02) was used to perform statistical analysis. The Kolmogorov–Smirnov test was used to verify the normal distribution of data from the RNAi assays (mRNA expression, ASFV genome copy number and virus titre) and differences between experimental groups were identified by the non-parametric Wilcoxon–Mann–Whitney test. *p-*values less than 0.05 were considered statistically significant.
